# Program for Reducing Obesity (PRO): An institutional review of an insurance‐based weight loss program utilizing shared medical appointments

**DOI:** 10.1002/osp4.564

**Published:** 2021-09-25

**Authors:** Jeffrey Wei, Lei Lei, Albert Shieh, Deepashree Gupta, Susan Ahern, Na Shen

**Affiliations:** ^1^ Department of Medicine Division of Endocrinology David Geffen School of Medicine at UCLA Los Angeles California USA; ^2^ Division of Geriatrics David Geffen School of Medicine at UCLA Los Angeles California USA

**Keywords:** insurance‐based, obesity, shared medical appointments, weight management

## Abstract

**Background:**

Based on CDC estimates in the United States, the prevalence of obesity was 42.4% in 2017–2018, and the annual cost of obesity was $147 billion in 2008. Yet studies estimate that only 20–40% of adults with obesity received counseling from their primary care providers. Recent studies using shared medical appointments (SMA), where patients are seen by a multidisciplinary team, have shown promising results in obesity management. We developed an insurance‐based weight loss program incorporating SMA, called the Program for Reducing Obesity (PRO), and report our findings here.

**Methods:**

Enrollment began in January 2019 at the UCLA Health Thousand Oaks clinic. Patients age ≥18 years with BMI ≥30 kg/m^2^ were eligible by referral to PRO, a program consisting of individual visits and SMAs with an obesity medicine board certified endocrinologist and registered dietitian. Primary outcomes were change in weight after 3, 6, and 12 months. Secondary outcomes included proportion that achieved ≥5% weight loss, change in percent body fat, HbA1c, HDL, triglycerides, and blood pressure.

**Results:**

102 patients (mean age 59.7 years, 72% women, mean weight 103.6 kg, mean BMI 36.6 kg/m^2^) have been analyzed, with 91 patients completing at least 12 months of the program. Patients achieved significant weight loss: 3.0%, 5.0%, and 7.8% of their baseline weight after 3, 6, and 12 months respectively. 52% of patients lost ≥5% of their baseline weight after 12 months. Patients had significant reductions in body fat: 2.1%, 7.4%, and 6.7% of their baseline (all *p* ≤ 0.01) after 3, 6, and 12 months respectively. Improvements were also seen in HbA1c (*p* ≤ 0.01), triglycerides (*p* ≤ 0.04), and systolic blood pressure (*p* ≤ 0.07) after 12 months although not all results achieved statistical significance.

**Conclusion:**

Our institutional review of PRO, an insurance‐based obesity program utilizing SMA, demonstrates a successful approach to promoting weight loss in a community‐based setting.

## 1 INTRODUCTION

Obesity as a chronic disease has risen to pandemic proportions over the last few decades.^1^ It is linked to an increased risk of various comorbidities^2^, ^3^, ^4^, ^5^ and significantly higher all‐cause mortality,^6^ and exacts a heavy financial burden on an individual and a national level.^7^, ^8^ The detrimental consequence of obesity is highlighted in the coronavirus disease 2019 (COVID‐19) pandemic. It is estimated that 30.2% of COVID‐19 hospitalizations were attributable to obesity alone.^9^

The foundations of obesity management are lifestyle modification and adjuvant pharmacotherapies. Despite the efficacy of lifestyle modification in obesity management,^10^, ^11^ practically it is challenging to provide intensive, frequent, individualized counseling during routine office visits. Providers often face a multitude of barriers, such as competing demands, time restraints, limited training, and a lack of financial incentive.^12^ Currently there are only a handful of FDA‐approved anti‐obesity medications (AOM) on the market, yet they remain grossly underutilized, as evidenced by the observation that only 0.8% eligible patients received AOM between 2015 and 2018 based on data from the National Health and Nutrition Examination Survey and Medical Expenditure Panel Survey.^13^ Less than a quarter of prescribing providers accounted for nearly 90% of all AOM prescriptions.^14^ The majority of AOM prescriptions came from Family Medicine/General Practice and Internal Medicine, while endocrinology had the highest prevalence of prescribers of all subspecialties.^15^ However this landscape may change with the recent availability of highly efficacious medications like the high dose semaglutide which has shown to result in as much as 16% weight loss compared to placebo.^16^ More weight loss programs involving endocrinologists will likely improve utilization of AOMs as well.

In recent years, shared medical appointments (SMA) have emerged as a potential option for efficient delivery of obesity care. SMAs allow patients sharing a common illness to convene as a group with one or more health care providers for education and counseling, clinical support, as well as individualized care including physical exam and medication management.^17^ SMAs improve care delivery and outcomes for patients and providers through fostering camaraderie amongst patients, facilitating knowledge‐sharing through peer‐to‐peer interaction, and building equitable relationship and trust between patients and providers.^17^

Utilization of SMA in obesity management has not been extensively studied. Previous studies show that patients attending SMAs were more likely to be prescribed anti‐obesity medications^18^ and to achieve sustained significant weight loss after 9 months,^19^ 12 months,^20^ and 24 months.^18^ SMAs were estimated to be four times more cost effective and seven times more time efficient than individual weight loss consultations.^20^ In patients with prediabetes, the implementation of SMA led to a modest mean weight loss of 6.6 pounds at 1 year as well as a modest drop in fasting blood glucose of 6 mg/dL.^21^ Of the previously mentioned studies, only Shibuya et al. focused on the impact of SMA programs led by endocrinologists. Given the significant overlap in disease burden of obesity and various endocrinopathies including diabetes, endocrinologists are well positioned to lead the effort to tackle the growing obesity pandemic. Here, we present our institutional findings of an insurance‐based weight loss program called the Program for Reducing Obesity (PRO), which incorporates repeating cycles of SMA sessions led by a dietician and an endocrinologist who is also board‐certified in obesity medicine. Our data will augment the growing literature of SMA in the management of obesity.

## 2 MATERIALS AND METHODS

### 2.1 Study design and population

Patients age ≥18 years with body mass index (BMI) ≥30 kg/m^2^ were eligible by referral from their UCLA primary care provider or endocrinologist to the Program for Reducing Obesity (PRO) at the UCLA Thousand Oaks clinic starting in January 2019. There were otherwise no explicit exclusion criteria. We analyzed the first 102 patients that were referred between January 2019 and August 2019. PRO continues to actively accept referrals for patients interested in joining the program. Thus, our cohort continues to grow beyond the initial 102 patients analyzed.

PRO is an insurance‐based program consisting of individual visits and shared medical appointments (SMA) with an obesity medicine board certified endocrinologist and registered dietitian.

Patients referred to the program initially met one on one for a 40 min visit with a PRO physician (an obesity medicine board certified endocrinologist) who then introduced them to the program, gathered pertinent past medical history, assessed risk factors for obesity and its comorbidities, obtained body composition data, formulated a nutrition and exercise plan, and completed a workup for secondary causes of obesity if indicated. These individualized visits with the physician occurred at minimum after 1 month, 3, 6 and 12 months after the initial visit. These patients were then referred to the optional weekly SMA visits where they received individual medical counseling from the PRO physician and nutritional counseling from the dietician in the form of group classes. In these two‐hour SMA visits, patients had their vitals taken, received pertinent clinical exams, had medication adjustments if indicated, and received group nutrition didactics and counseling under the guidance of both a PRO physician and registered dietician. All individual and SMA visits were in person except for the period of March through August of 2020 where we transitioned to telehealth visits due to the pandemic.

### 2.2 Measures

Primary outcomes were absolute and percent change in weight and BMI after 3, 6, and 12 months from baseline. Baseline weight was defined as the weight measured at the initial visit for PRO. Subsequent weight measurements at 3, 6, and 12 months were obtained from either PRO appointments or at other UCLA clinic visits.

Secondary outcomes included proportion that achieved weight loss and ≥5% weight loss, change in percent body fat, hemoglobin A1c (HbA1c), high‐density lipoprotein (HDL), triglycerides, systolic blood pressure (SBP), and diastolic blood pressure (DBP). Percent body fat was obtained using a Tanita segmental body composition analyzer. The Tanita segmental body composition analyzer uses bioelectric impedance to measure body composition.

Additional information, including age at start of program, race, ethnicity, number of visits with the obesity medicine board certified endocrinologist, number of nutrition classes attended, history of bariatric surgery, history of diabetes and prediabetes, and proportion that were prescribed weight loss medications were collected. Weight loss medications included the following: phentermine, topiramate, phentermine‐topiramate, lorcaserin, naltrexone‐bupropion, diethylpropion, and liraglutide. After lorcaserin was withdrawn from the market in February 2020, patients in the program that were prescribed lorcaserin were recommended to discontinue this medication.

### 2.3 Statistical analysis

Descriptives for all variables were generated, and assessed for normality. Absolute and percent change in each variable relative to baseline were calculated for 3, 6, and 12 months. We used the paired *t*‐test to compare two groups and ANOVA to compare more than two groups to determine whether change from baseline was significant at a two‐sided alpha of 0.05. All statistical analyses were performed using STATA 14.

## 3 RESULTS

### 3.1 Study participants

One hundred and two patients were analyzed in this report, with 91 patients completing at least 12 months of the program. Demographics and baseline characteristics are shown in Table 1. Most participants were female (72%) and Caucasian (80%). Mean age was 59.7 years with a mean weight of 103.6 kg, mean BMI of 36.6 kg/m^2^, and mean percent body fat of 42.6%. 70% of patients attended at least one nutrition class. 59% of patients were prescribed a weight loss medication during the program.

**TABLE 1:** Baseline demographics

Demographics	*N* = 102
Age, years (mean ± SD)	59.7 ± 13.3
Sex female, *n* (%)	73 (72)
Race, *n* (%)	
Caucasian	82 (80)
Asian	4 (4)
Black	2 (2)
Other	9 (9)
Declined/Unknown	4 (5)
Ethnicity, *n* (%)	
Hispanic	7 (7)
Not Hispanic	86 (84)
Declined/Unknown	9 (9)
Appointments with physician, *n* (mean ± SD)	5.0 ± 2.4
PRO nutrition group classes attended, *n* (mean ± SD)	3.5 ± 5.7
Took at least 1 PRO nutrition group class, *n* (%)	71 (70)
History of bariatric surgery, *n* (%)	4 (4)
History of diabetes, *n* (%)	33 (32)
History of prediabetes, *n* (%)	47 (46)
Taking weight loss medications, *n* (%)	60 (59)
Weight, kg (mean ± SD)	103.6 ± 21.5
BMI, kg/m^2^ (mean ± SD)	36.6 ± 6.3
Percent body fat, % (mean ± SD) (*n* = 69)	42.6 ± 8.4
HbA1c, % (mean ± SD) (*n* = 81)	6.3 ± 1.1
SBP, mm Hg (mean ± SD)	130 ± 14
DBP, mm Hg (mean ± SD)	77 ± 8
Triglycerides, mg/dl (mean ± SD) (*n* = 70)	146.9 ± 172.8
HDL, mg/dl (mean ± SD) (*n* = 70)	52.4 ± 14.1

Abbreviations: BMI, body mass index; DBP, diastolic blood pressure; HbA1c, hemoglobin A1c; HDL, high‐density lipoprotein; PRO, program for reducing obesity; SBP, systolic blood pressure.

### 3.2 Primary outcomes

**FIGURE 1:**
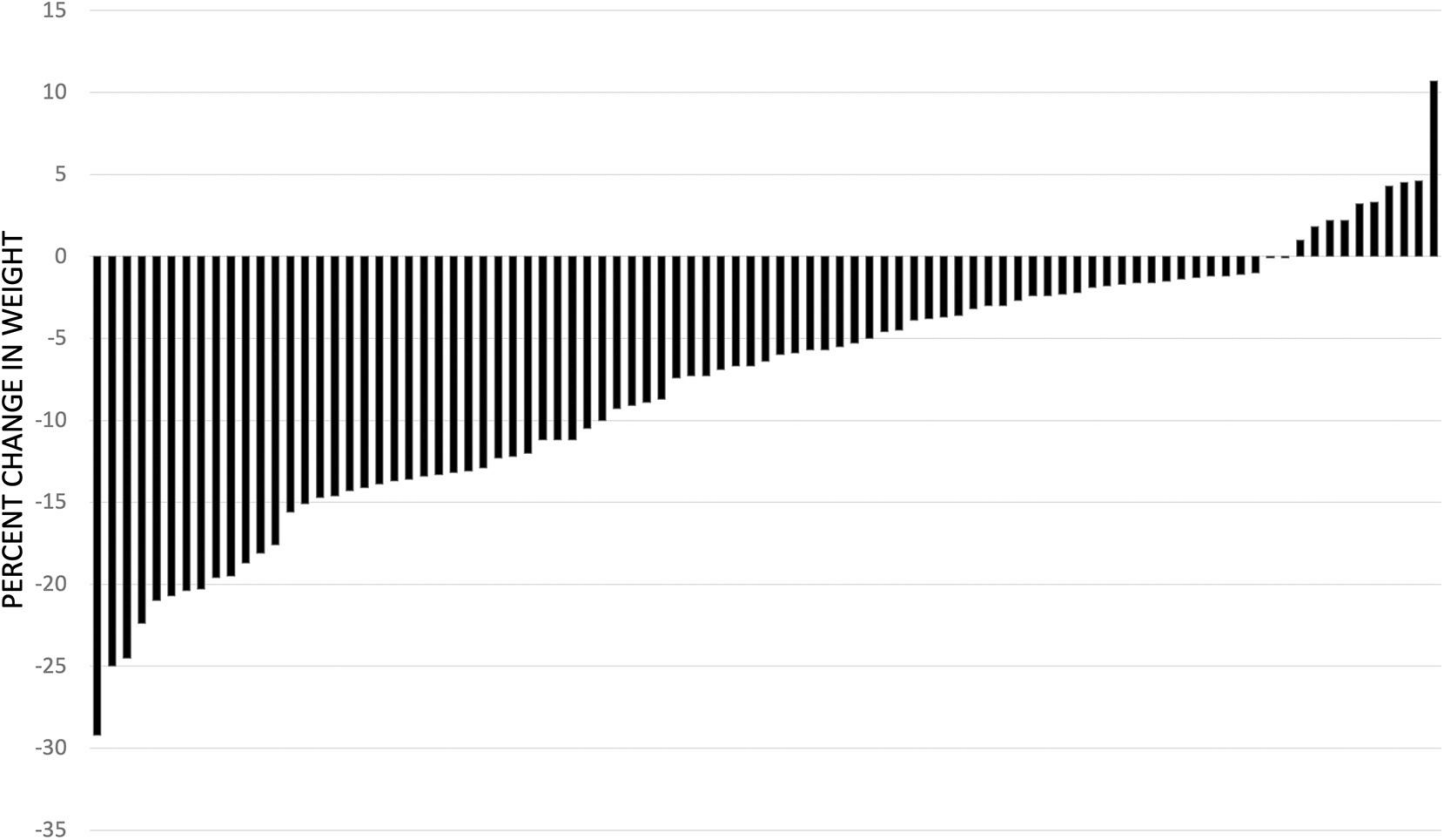
Individual weight change in 12 months

Primary outcomes are reported in Table 2. Patients achieved statistically significant weight loss: 3.0% (3.1 kg), 5.0% (5.1 kg), and 7.8% (8.4 kg) of their baseline weight after 3, 6, and 12 months respectively (*p*‐values <0.0001 for all comparisons). Similarly, patients achieved statistically significant reductions in BMI: 2.9% (1.1 kg/m^2^), 5.1% (1.9 kg/m^2^), and 7.9% (3.0 kg/m^2^) of their baseline BMI after 3, 6, and 12 months respectively (*p*‐values <0.0001 for all comparisons). Figure 1 shows the percent weight change from baseline of each patient who completed at least 12 months of the program. The percent weight change at 12 months ranged from −29.2% to 10.7%.

**TABLE 2:** Primary outcomes

		Average	Absolute change from baseline	Percent change from baseline	*N*	*p*‐value
Weight, kg (mean ± SD)	Baseline	103.6 ± 21.5	–	–	102	–
	3 months	100.5 ± 21.4	−3.1	−3.0	96	<0.0001
	6 months	98.5 ± 21.0	−5.1	−5.0	93	<0.0001
	12 months	95.9 ± 20.2	−8.4	−7.8	91	<0.0001
BMI, kg/m^2^ (mean ± SD)	Baseline	36.6 ± 6.3	–	–	102	–
	3 months	35.5 ± 6.3	−1.1	−2.9	96	<0.0001
	6 months	34.8 ± 6.1	−1.9	−5.1	93	<0.0001
	12 months	33.9 ± 6.0	−3.0	−7.9	91	<0.0001

Abbreviation: BMI, body mass index.

### 3.3 Secondary outcomes

Secondary outcomes are reported in Tables 3a and 3b. 79.4% of patients achieved weight loss at 12 months 52.0% of patients lost at least 5% of their baseline weight after 12 months. Patients had significant reductions in body fat: 2.1%, 7.4%, and 6.7% of their baseline (all *p* ≤ 0.01) after 3, 6, and 12 months respectively. Improvements were also seen in HbA1c (*p* ≤ 0.01), triglycerides (*p* ≤ 0.04), and systolic blood pressure (*p* ≤ 0.07) after 12 months although not all results achieved statistical significance.

**TABLE 3a:** Secondary outcomes

Achieved weight loss, %	3 months	76.5
	6 months	79.4
	12 months	79.4
Achieved ≥5% weight loss, %	3 months	31.4
	6 months	41.2
	12 months	52.0

**TABLE 3b:** Secondary outcomes

		Average	Absolute change from baseline	Percent change from baseline	*N*	*p*‐value
Percent body fat, % (mean ± SD)	Baseline	42.6 ± 8.4	–	–	69	–
	3 months	41.7 ± 7.6	−0.9	−2.1	55	0.01
	6 months	39.4 ± 8.6	−3.1	−7.4	52	<0.0001
	12 months	39.9 ± 7.4	−3.0	−6.7	35	0.0003
HbA1c, % (mean ± SD)	Baseline	6.3 ± 1.1	–	–	81	–
	3 months	6.3 ± 0.9	−0.002	−0.03	50	0.01
	6 months	6.1 ± 0.8	−0.2	−3.1	70	0.0003
	12 months	6.2 ± 0.9	−0.2	−3.2	44	0.01
Triglycerides, mg/dl (mean ± SD)	Baseline	146.9 ± 172.8	–	–	70	–
	3 months	139.6 ± 74.0	−7.3	−5.0	47	0.3
	6 months	134.0 ± 69.4	−12.9	−8.8	64	0.2
	12 months	126.1 ± 70.3	−15.0	−7.8	34	0.04
HDL, mg/dl (mean ± SD)	Baseline	52.4 ± 14.1	–	–	70	–
	3 months	50.6 ± 11.5	−1.8	−3.3	47	0.3
	6 months	51.0 ± 12.6	−1.4	−2.6	64	0.5
	12 months	51.5 ± 14.1	−0.1	−1.4	34	0.9
SBP, mm Hg (mean ± SD)	Baseline	130 ± 14	–	–	102	–
	3 months	128 ± 13	−2.6	−2.0	98	0.04
	6 months	129 ± 16	−1.5	−1.1	95	0.2
	12 months	127 ± 15	−3.3	−1.7	86	0.07
DBP, mm Hg (mean ± SD)	Baseline	77 ± 8	–	–	102	–
	3 months	77 ± 8	−0.6	−0.8	98	0.3
	6 months	76 ± 9	−1.5	−2.0	95	0.1
	12 months	76 ± 8	−1.3	−0.6	86	0.2

Abbreviations: DBP, diastolic blood pressure; HbA1c, hemoglobin A1c; HDL, high‐density lipoprotein; SBP, systolic blood pressure.

### 3.4 Supplementary analysis

Supplementary analysis was performed to explore the change in BMI at 12 months from baseline, stratified by use of weight loss medications and weekly SMA group nutrition classes. There was a trend for greater reductions in BMI after 12 months in patients who both took weight loss medications and attended weekly SMAs, but the differences were not statistically significant (*p* ≤ 0.08, Table S1). There was a trend showing greater reductions in BMI after 12 months for patients with a higher initial obesity class, however this was not statistically significant (data not shown).

## 4 DISCUSSION

In our institutional review of an insurance‐based obesity program utilizing SMA, we found statistically significant weight loss for patients after 3, 6, and 12 months. At 12 months, over half of the patients achieved at least 5% of weight loss compared to baseline weight. A 5% reduction in weight is clinically important due to associated improvements in multiple comorbidities, including prevention of diabetes, glycemic control in patients with diabetes, hyperlipidemia, hepatic steatosis, sleep apnea, knee pain, depression, sexual function, morbidity, and mortality.^22^ Moreover, a reduction in BMI by 5%–10% has been associated with substantial savings in annual medical expenditures.^23^

Our findings are consistent with prior studies implementing SMAs for weight management.^18^, ^19^, ^20^, ^24^ A longitudinal retrospective cohort study comparing patients who attended SMAs (n = 310) to patients provided usual care (n = 1993) showed statistically significant weight loss of 5.2% in the SMA group versus 1.8% in the non‐SMA group after one year.^18^ A non‐randomized two‐year study comparing patients who attended SMAs (n = 74) to patients provided usual care (n = 356) during the same period showed modest but statistically significant weight loss of 1.0% in the SMA group versus weight gain of 0.8% in the non‐SMA group.^24^ A single‐arm study following 216 patients who attended SMAs showed statistically significant weight loss of 3.2% after one year compared to baseline, and in those who attended at least four SMAs, average weight loss improved to 4.3% after one year.^20^ A retrospective observational study following 222 patients who attended SMAs showed that 41% of patients achieved 5% weight loss at 9 months, with significant reductions in blood pressure and HbA1c.^19^ We also demonstrate a concurrent reduction in body fat, which is an independent risk factor for metabolic dysfunction and obesity‐related comorbidities.^25^

There are limitations of our study. This study was non‐controlled and retrospective in nature. Most participants in the program were female and Caucasian, and thus results may not be generalizable. The frequency of individual follow‐up visits and group nutrition classes was left at the discretion of the provider and patient, which introduced heterogeneity of patient care. For example, roughly 30% of patients did not attend the optional weekly SMAs. This was due to either patient preference, work schedule, or other prior commitments. The supplementary analyses suggest a trend of more weight loss with attendance of weekly SMAs and use of weight loss medications, however this was not statistically significant, and may be a reflection of a small sample size. Not requiring attendance of SMAs may have limited or attenuated our primary and secondary outcomes. However, this heterogeneity of patient care may reflect a more practical and patient‐centered quality of care. Although certain weight loss medications were indicated based on the patients' BMI, insurances may not have provided adequate financial coverage for these medications, which added an additional barrier to optimizing patient care. There are missing outcome measurements, particularly with regards to the secondary outcomes, as a result of this study being retrospective in nature. Thus, the comparisons from one time point to baseline for certain measurements are limited and should be interpreted with caution.

The COVID‐19 pandemic also brought about additional challenges wherein the weekly SMA nutrition group classes were transitioned to video conferencing as large group sessions were not allowed. Most patients opted to utilize telemedicine for their visits with the obesity medicine board certified endocrinologist, particularly during the height of the pandemic. The impact of the pandemic may have placed additional stressors on patients' ability to adhere to a healthy diet and an active lifestyle, and thus, may have influenced our primary and secondary outcomes.

As PRO continues to expand and evolve, we have identified opportunities for improvements to the program. As the number of referrals increased, our SMA group classes exceeded capacity, so we have added concurrent weekly SMA group classes each week to help increase attendance and availability. We have also started monthly SMA group classes for patients who have achieved their weight loss goals and are being followed for weight maintenance.

This study supports findings from similar studies showing that the SMA model is an efficient and effective weight loss option for patients. Our program is unique in that we utilize both individual visits with an obesity board certified endocrinologist in addition to SMAs, which may explain the higher percentage of weight loss seen here compared with other SMA studies. Furthermore, an insurance‐based weight management program like PRO can serve as a cost‐effective model for community clinics to address the growing obesity epidemic. Future studies will explore whether the frequency of provider visits and weekly SMA group classes have an impact on the amount of weight change. We also plan to conduct an extension to our study to assess whether these patients are able to maintain weight loss in the long term.

## 5 CONCLUSION

Our institutional review of PRO, an all insurance‐based obesity program utilizing SMA demonstrates a successful approach to promoting weight loss in a community‐based setting.

## CONFLICT OF INTEREST

The authors declare no conflicts of interest.

## AUTHOR CONTRIBUTION

Dr. Wei carried out the majority of the research project and the manuscript writing. Dr. Lei helped with data collection for the research project and assisted in manuscript writing. Dr. Shieh performed all the statistical analysis for the research project and assisted in manuscript writing. Dr. Gupta assisted in data collection for the research project and contributed input to the manuscript writing. Dr. Ahern performed research for the project and contributed input to the manuscript writing. Dr. Shen designed and directed the research project as well as supervised the manuscript writing.

## Supporting information

Supplementary Material

## References

[1] Hales CM , Carroll MD , Fryar CD , Ogden CL . Prevalence of obesity and severe obesity among adults: United States, 2017–2018. NCHS Data Brief. 2020;(360):1‐8. 32487284

[2] Engin A . The definition and prevalence of obesity and metabolic syndrome. Adv Exp Med Biol. 2017;960:1‐17 doi:10.1007/978‐3‐319‐48382‐5_1 28585193 10.1007/978-3-319-48382-5_1

[3] Wiebe N , Stenvinkel P , Tonelli M . Associations of chronic inflammation, insulin resistance, and severe obesity with mortality, myocardial infarction, cancer, and chronic pulmonary disease. JAMA Netw Open. 2019;2(8):e1910456. doi:10.1001/jamanetworkopen.2019.10456 31469399 10.1001/jamanetworkopen.2019.10456 PMC6724168

[4] Jehan S , Myers AK , Zizi F , Pandi‐Perumal SR , Jean‐Louis G , McFarlane SI . Obesity, obstructive sleep apnea and type 2 diabetes mellitus: Epidemiology and pathophysiologic insights. Sleep Med Disord. 2018;2(3):52‐58. 30167574 PMC6112821

[5] Yarnoff BO , Hoerger TJ , Shrestha SS , et al. Modeling the impact of obesity on the lifetime risk of chronic kidney disease in the United States using updated estimates of GFR progression from the CRIC study. PLoS One. 2018;13(10):e0205530. doi:10.1371/journal.pone.0205530 30339684 10.1371/journal.pone.0205530 PMC6195263

[6] Flegal KM , Kit BK , Orpana H , Graubard BI . Association of all‐cause mortality with overweight and obesity using standard body mass index categories: A systematic review and meta‐analysis. JAMA. 2013;309(1):71‐82. doi:10.1001/jama.2012.113905.PMID:23280227 23280227 10.1001/jama.2012.113905 PMC4855514

[7] Apovian CM . Obesity: Definition, comorbidities, causes, and burden. Am J Manag Care. 2016;22(7 Suppl):s176‐85. 27356115

[8] Kim DD , Basu A . Estimating the medical care costs of obesity in the United States: Systematic review, meta‐analysis, and empirical analysis. Value Health. 2016;19(5):602‐13. doi:10.1016/j.jval.2016.02.008 27565277 10.1016/j.jval.2016.02.008

[9] O'Hearn M , Liu J , Cudhea F , Micha R , Mozaffarian D . Coronavirus disease 2019 hospitalizations attributable to cardiometabolic conditions in the United States: A comparative risk assessment analysis. J Am Heart Assoc. 2021;10(5):e019259. doi:10.1161/JAHA.120.019259 33629868 10.1161/JAHA.120.019259 PMC8174244

[10] Diabetes Prevention Program (DPP) Research Group . The Diabetes prevention program (DPP): Description of lifestyle intervention. Diabetes Care2002;25:2165‐71. doi:10.2337/diacare.25.12.2165pmid:12453955 10.2337/diacare.25.12.2165 PMC1282458 12453955

[11] Look AHEAD Research Group , Wadden TA , West DS , et al. The Look AHEAD study: A description of the lifestyle intervention and the evidence supporting it. Obesity (Silver Spring). 2006;14(5):737‐52. doi:10.1038/oby.2006.84 16855180 10.1038/oby.2006.84 PMC2613279

[12] Haire‐Joshu D , Klein S . Is primary care practice equipped to deal with obesity? Comment on "preventing weight gain by lifestyle intervention in a general practice setting". Arch Intern Med. 2011;171(4):313‐5. doi:10.1001/archinternmed.2011.3 21357806 10.1001/archinternmed.2011.3 PMC3607436

[13] MacEwan J , Kan H , Chiu K , Poon JL , Shinde S , Ahmad NN . Anti‐obesity medication use among adults with overweight and obesity in the United States: 2015–2018. Endocr Pract. 2021:S1530‐891X(21)01122‐8. doi:10.1016/j.eprac.2021.07.004 10.1016/j.eprac.2021.07.004 34265455

[14] Saxon DR , Iwamoto SJ , Mettenbrink CJ . Antiobesity medication use in 2.2 million adults across eight large health care organizations: 2009–2015. Obesity (Silver Spring). 2019;27(12):1975‐1981. doi:10.1002/oby.22581 31603630 10.1002/oby.22581 PMC6868321

[15] Thomas CE , Mauer EA , Shukla AP , Rathi S , Aronne LJ . Low adoption of weight loss medications: A comparison of prescribing patterns of antiobesity pharmacotherapies and SGLT2s. Obesity (Silver Spring). 2016;24(9):1955‐61. doi:10.1002/oby.21533 27569120 10.1002/oby.21533 PMC5669035

[16] Wadden TA , Garvey T , Bailey TS , et al. Effect of subcutaneous semaglutide vs placebo as an adjunct to intensive behavioral therapy on body weight in adults with overweight or obesity: The step 3 randomized clinical trial. JAMA. 2021;325(14):1403‐1413. 33625476 10.1001/jama.2021.1831 PMC7905697

[17] Kirsh SR , Aron DC , Johnson KD , et al. A realist review of shared medical appointments: How, for whom, and under what circumstances do they work? BMC Health Serv Res. 2017;17(1):113. doi:10.1186/s12913‐017‐2064‐z 28160771 10.1186/s12913-017-2064-z PMC5291948

[18] Shibuya K , Ji X , Pfoh ER , et al. Association between shared medical appointments and weight loss outcomes and anti‐obesity medication use in patients with obesity. Obes Sci Pract. 2020;6(3):247‐254. doi:10.1002/osp4.406 32523713 10.1002/osp4.406 PMC7278906

[19] Yager S , Parker M , Luxenburg J , Varghai NH . Evaluation of multidisciplinary weight loss shared medical appointments. J Am Pharm Assoc (2003). 2020;60(1):93‐99. doi:10.1016/j.japh.2019.07.014 31466900 10.1016/j.japh.2019.07.014

[20] Egger G , Stevens J , Volker N , Egger S . Programmed shared medical appointments for weight management in primary care: An exploratory study in translational research. Aust J Gen Pract. 2019;48(10):681‐688. doi:10.31128/AJGP‐05‐19‐4940 31569313 10.31128/AJGP-05-19-4940

[21] Cole RE , Boyer KM , Spanbauer SM , Sprague D , Bingham M . Effectiveness of prediabetes nutrition shared medical appointments: Prevention of diabetes. Diabetes Educ. 2013;39(3):344‐53. doi:10.1177/0145721713484812 23589326 10.1177/0145721713484812

[22] Ryan DH , Yockey SR . Weight loss and improvement in comorbidity: Differences at 5%, 10%, 15%, and Over. Curr Obes Rep. 2017;6(2):187‐194. doi:10.1007/s13679‐017‐0262‐y 28455679 10.1007/s13679-017-0262-y PMC5497590

[23] Cawley J , Meyerhoefer C , Biener A , Hammer M , Wintfeld N . Savings in medical expenditures associated with reductions in body mass index among US adults with obesity, by diabetes status. Pharmacoeconomics. 2015;33(7):707‐22. doi:10.1007/s40273‐014‐0230‐2 25381647 10.1007/s40273-014-0230-2 PMC4486410

[24] Palaniappan LP , Muzaffar AL , Wang EJ , Wong EC , Orchard TJ . Shared medical appointments: promoting weight loss in a clinical setting. J Am Board Fam Med. 2011;24(3):326‐8. doi:10.3122/jabfm.2011.03.100220 21551406 10.3122/jabfm.2011.03.100220 PMC3217311

[25] Frank AP , de Souza Santos R , Palmer BF , Clegg DJ . Determinants of body fat distribution in humans may provide insight about obesity‐related health risks. J Lipid Res. 2019;60(10):1710‐1719. doi:10.1194/jlr.R086975 30097511 10.1194/jlr.R086975 PMC6795075

