# Supplementary material for: Program for Reducing Obesity (PRO): An institutional review of an insurance‐based weight loss program utilizing shared medical appointments
Source: Obes Sci Pract. 2021 Sep 25;8(3):272–8. doi: 10.1002/osp4.564 (PMC9159562; doi:10.1002/osp4.564)
Supplement: Supplementary file 1 — Supplementary Material [file OSP4-8-272-s001.docx]

Supplementary Table 1: Subanalyses of primary outcomes, stratified by use of weight loss medications and/or attendance of weekly SMA nutrition group classes

|  | AOM=No  SMA=Yes  (n=30) | AOM=Yes  SMA=No  (n=21) | AOM=Yes  SMA=Yes  (n=31) | P-value |
| --- | --- | --- | --- | --- |
| Absolute Change in BMI at 12 months from Baseline, kg/m^2^ | -2.4 ± 3.0 | -2.8 ± 3.3 | -3.9 ± 3.4 | 0.16 |
| Percent Change in BMI at 12 months from Baseline, % | -6.1 ± 7.6 | -7.0 ± 7.1 | -10.5 ± 8.6 | 0.08 |
| AOM = Took weight loss medications (anti-obesity medications)  SMA = Attended weekly SMA nutrition group classes  BMI = body mass index | | | | |
